# Serum Elabela expression is decreased in hypertensive patients and could be associated with the progression of hypertensive renal damage

**DOI:** 10.1186/s40001-024-01674-1

**Published:** 2024-02-01

**Authors:** Geng Tian, Qian Zheng, Qingru Zhang, Xiaoyu Liu, Xuehong Lu

**Affiliations:** 1 Second Hospital of Jilin University, Changchun, 130041 China; 2 Jiading District Central Hospital Affiliated Shanghai University of Medicine &Health Sciences, Shanghai, 201800 China; 3 Department of Nephrology, Second Hospital, Jilin University, 218 Ziqiang Street, Changchun, 130041 Jilin China

**Keywords:** eGFR, Elabela, Hypertension, Malignant hypertension, Renal damage, Systolic blood pressure

## Abstract

**Background:**

Elabela, a recently discovered hormonal peptide containing 32 amino acids, is a ligand for the apelin receptor. It can lower blood pressure and attenuate renal fibrosis. However, the clinicopathological relationship between Elabela level and renal damage caused by benign hypertension (BHT) and malignant hypertension (MHT) has not been elucidated. Therefore, we investigated the clinicopathological correlation between serum Elabela level and renal damage caused by BHT and MHT.

**Methods:**

The participants comprised 50 patients and 25 age-matched healthy adults. The 50 patients were separated into two groups: MHT (*n* = 25) and BHT groups (*n* = 25). We analyzed their medical histories, demographics, and clinical examinations, including physical and laboratory tests.

**Results:**

The results showed that serum Elabela level decreased gradually with a continuous increase in blood pressure from the healthy control group, BHT, to MHT. Moreover, Elabela levels negatively correlated with BMI (*R* =  − 0.27, *P* = 0.02), SBP (*r* =  − 0.64, *P* < 0.01), DBP (*r* =  − 0.58, *P* < 0.01), uric acid (*r* =  − 0.39, *P* < 0.01), bun (*r* =  − 0.53, *P* < 0.01), and Scr (*r* =  − 0.53 *P* < 0.01) but positively correlated with eGFR (*r* = 0.54, *P* < 0.01). Stepwise multivariate linear regression analysis showed that SBP was the variable most related to Elabela (*t* =  − 5.592, *P* < 0.01).

**Conclusions:**

Serum Elabela levels decreased in patients with hypertension, especially malignant hypertension, and has the potential to be a marker of hypertension-related kidney damage.

## Background

Hypertension, one of the most common chronic diseases worldwide, is a risk factor for kidney diseases [1]. The prevalence of hypertension is gradually increasing in China. The results of the 2012–2015 survey using a stratified multistage random sampling method to obtain a nationally representative sample of 451 755 residents ≥ 18 years of age from 31 provinces in Mainland China showed that the prevalence rate of hypertension among the Chinese adult population was approximately 23.2% [2]. Malignant hypertension (MHT), also known as accelerated-malignant hypertension or malignant-phase hypertension, is the most serious form of hypertension and is a systemic disease characterized by extremely elevated systolic blood pressure and out-of-range office diastolic blood pressure [above 130 mmHg] at the time of diagnosis as well as acute ischemic organ damage (kidney, eye, etc.) [3–5]. Although the diagnosis of MHT is simple, clinically, it can only be diagnosed when the target organs are damaged [6], and the kidney is the organ most commonly affected by hypertension-mediated damage, and the damage to the kidney is irreversible. Therefore, early diagnosis and treatment of MHT are critical for maintaining the function of hypertension-mediated damaged kidney [4, 7]. 

The apelin receptor (APJ), also known as an angiotensin-like receptor, is a G protein-coupled receptor [8]. In recent years, two independent studies have discovered and reported a homologous ligand of the APJ, Elabela, which consists of a conserved sequence of 54 amino acids, including a secretory signal with a mature hormonal peptide containing 32 amino acids [9, 10]. Elabela is highly expressed during the embryonic stage and contributes to cardiac morphogenesis, endoderm differentiation, mesoderm cell movement, and neovascularization [11–13]. In adulthood, Elabela is primarily expressed in the kidney and in mice, and it relaxes constricted aortic blood vessels by binding to the APJ [14, 15]. Previous studies have shown that Elabela plays an anti-hypertensive role in hypertensive nephropathy and alleviates fibrosis [16].

However, the clinicopathological relationship between Elabela level and renal damage caused by benign hypertension (BHT) and MHT has not been elucidated. Therefore, this study aimed to explore Elabela as a biomarker to predict the extent of hypertension-associated kidney damage.

## Methods

### Study participants and groups

We recruited 50 patients with MHT and BHT diagnosed by renal biopsy diagnosis, as well as 25 healthy normotensive participants, from January 2016 to November 2021. The participants underwent a full medical history and clinical examination, including physical and laboratory tests. According to the 2018 European Society of Cardiology (ESC)/European Society of Hypertension (ESH) Guidelines for the Management of Arterial Hypertension: The Task Force for the Management of Arterial Hypertension of the ESC and ESH [17], patients with systolic blood pressure (SBP) ≥ 140 mmHg and/or diastolic blood pressure (DBP) ≥ 90 mmHg were enrolled. The twenty healthy normotensive participants had no kidney disease, had negative results on urinary dipstick analysis, and exhibited SBP ≤ 120 mmHg and DBP ≤ 80 mmHg in the absence of antihypertensive drugs. 

The clinical diagnosis of MHT was as follows: arterial blood pressure increased sharply in a short period, diastolic blood pressure ≥ 130 mmHg (or systolic blood pressure ≥ 180 mmHg), and fundus changes showed hypertensive retinal changes (retinal cotton exudation, hemorrhage, with or without optic papilla edema) [5]. The pathological diagnosis of renal damage in MHT was based on malignant hypertensive nephrosclerosis, characterized by proliferative arterioles (interlobular and arcuate arteries), endomyelitis (onion skin-like changes), and fibrinoid necrosis of the arteriole wall.

The exclusion criteria for the participants were as follows: (a) clinical evidence of diabetes mellitus, heart failure, myocardial infarction, peripheral artery disease, tumor, and hepatic or thyroid dysfunction; (b) renal replacement therapy, including hemodialysis, peritoneal dialysis, or renal transplantation; (c) pregnant women; (d) age ≥ 65 years or < 18 years; and (2) identification of other pathologies on renal biopsy.

The patients were partitioned into two groups according to the clinical diagnosis or pathological results of renal biopsy: MHT group (*n* = 25) and BHT group (*n* = 25). In addition to the patients, healthy controls were conducted, including 25 healthy normotensive participants. These groups were treated as disease controls and healthy controls, respectively.

### Laboratory parameters

The demographic data obtained were as follows: age, SBP, DBP, and body mass index (BMI). Fasting venous blood was collected to determine blood urea nitrogen (BUN), uric acid, serum creatinine (Scr), and estimated glomerular filtration rate (eGFR). The eGFR was calculated using the Chronic Kidney Disease Epidemiology Collaboration equation: eGFR > 30–59 mL/min/1.73 m^2^ indicates moderately decreased and eGFR < 30 mL/min/1.73 m^2^ indicates severely decreased [18].

For blood pressure measurements, three measurements were taken using a validated electronic upper-arm cuff or manual auscultatory after the participants had rested for 3–5 min. Briefly, the cuff was deflated at a speed of 2 mmHg/s and then phase I and V (disappearance) Korotkoff sounds were respectively used to identify SBP and DBP, taking the average of the last two blood pressure readings [17, 19].

In addition to the aforementioned parameters, the renal histopathological data of some patients were collected. Assessment of the renal tubulointerstitial damage in the renal cortex of patients employed a semi-quantitative scoring system based on Oxford’s research [20], tubular atrophy/interstitial fibrosis, and TAIF (0, absent; 1, ≤ 25%; 2, 26–50%; 3, > 50%). Both BHT and MHT were characterized by arterial or arteriolar hyalinosis and intimal fibrosis in renal tissue, whereas MHT was often accompanied by fibrinoid necrosis (acute stage) or myointimal cell proliferation (‘onion-skinning’ appearance, chronic stage). Vascular lesions were semi-quantitatively scored for intimal fibrosis on a scale of 0 to 2 (0, absent; 1, ≤ 50; 2, > 50%) [21, 22].

For determination of serum Elabela levels, venous blood was collected from the patients on the day of admission and from the healthy subjects into a gel separation vacuum tube containing heparin, EDTA, or citric acid as anticoagulant, and then centrifuged at 3000 rpm for 10 min. The supernatant was taken and stored in a refrigerator at − 80 ℃. The serum Elabela levels were determined using an ELABELA (human)-EIA kit (Peninsula Laboratories International, Inc., San Carlos, CA, USA), according to the manufacturer’s instructions.

### Statistical analysis

Statistical analyses were performed using the SPSS software (version 26.0; Chicago, IL, USA). The Shapiro–Wilk test was used to determine whether the distribution of continuous variables was normal. The continuous variables with normal distribution or approximate normal distribution were expressed as mean ± standard deviation, whereas those with skewed distributions were expressed as medians with 25th and 75th quartiles. The continuous variables were compared using the one-way analysis of variance, chi-square (*χ*^2^) test, or Kruskal–Wallis test. Multiple linear regression, Pearson correlation, and Spearman’s correlation analyses were used to identify correlations among the variables. Statistical significance was set at *P* < 0.05.

## Results

### Demographic and clinicopathological characteristics 

The characteristics of the participants are presented in Table 1. Age and gender were not significantly different among the three groups. Compared with the healthy control group, the two groups exhibited significantly higher SBP, DBP, uric acid, BUN, and Scr (*P* < 0.01), and appreciably lower eGFR (*P* < 0.01). Among the three groups, SBP, DBP, Uric acid, BUN, and Scr were the highest within the MHT group, whereas the eGFR was the lowest within the MHT group (Table 1).

**Table 1:** Demographic and clinicopathological data of all participants

	Healthy control (*n* = 25)	BHT (*n* = 25)	MHT (*n* = 25)	*P*
Gender (male/female)	19/6	21/4	24/1	0.10
Age (years)	42.48 ± 8.39	40.08 ± 10.43	36.32 ± 11.67	0.12
BMI (kg/m^2^)	23.07 ± 1.68	29.24 ± 4.80^c^	26.65 ± 4.40^a^	<0.01
SBP (mmHg)	118.6 ± 4.7	147.5 ± 9.4^c^	193.8 ± 19.0^a,b^	<0.01
DBP (mmHg)	79.88 ± 4.02	92.00 (90.00–100.00)^c^	114.36 ± 14.33^a,b^	<0.01
Uric acid	274.84 ± 59.69	503.92 ± 116.81^c^	508.56 ± 120.21^a^	<0.01
BUN (mmol/L)	5.17 ± 1.64	7.53 ± 3.10	15.52 (12.15–22.89)^a,b^	<0.01
Scr (μmol/L)	59.25 ± 11.37	132.00 (103.50–180.50)^c^	390.00 (237.00–546.50)^a,b^	<0.01
eGFR (mL/min/1.73 m^2^)	118.60 ± 22.74	57.67 ± 30.06^c^	17.00 (8.20–30.00)^a,b^	<0.01
Elabela (ng/ml)	6.29 ± 0.94	4.73 ± 1.25^c^	3.82 ± 1.08^a,b^	<0.01
Histopathological				
TAIF	–	1.88 ± 0.78	2.63 ± 0.71	<0.01
Intimal fibrosis	–	1.00(1.00, 2.00)	2.00(2.00, 2.00)	<0.01
Fibrinoid necrosis, n (%)	–	0	9 (62.5)	<0.01
Myointimal cell proliferation, n (%)	–	0	22 (91.7)	<0.01

*SBP* systolic blood pressure, *DBP* diastolic blood pressure, *BUN* blood urine nitrogen, *Scr* serum creatinine, *eGFR* estimated glomerular filtration rate, *TAIF* tubular atrophy/interstitial fibrosis.

^a^*P* < 0.05, healthy control *vs.* MHT group; ^b^*P* < 0.05, BHT group *vs.* MHT group; ^c^*P* < 0.05, healthy control *vs.* BHT group

Tubular atrophy/interstitial fibrosis and intimal fibrosis were more significant in MHT patients than in BHT patients (*P* < 0.05) (Table 1). Myointimal cell proliferation (91.7%) and fibrinoid necrosis (62.5%) were only observed in the MHT group.

### Serum Elabela levels

**Fig. 1:**
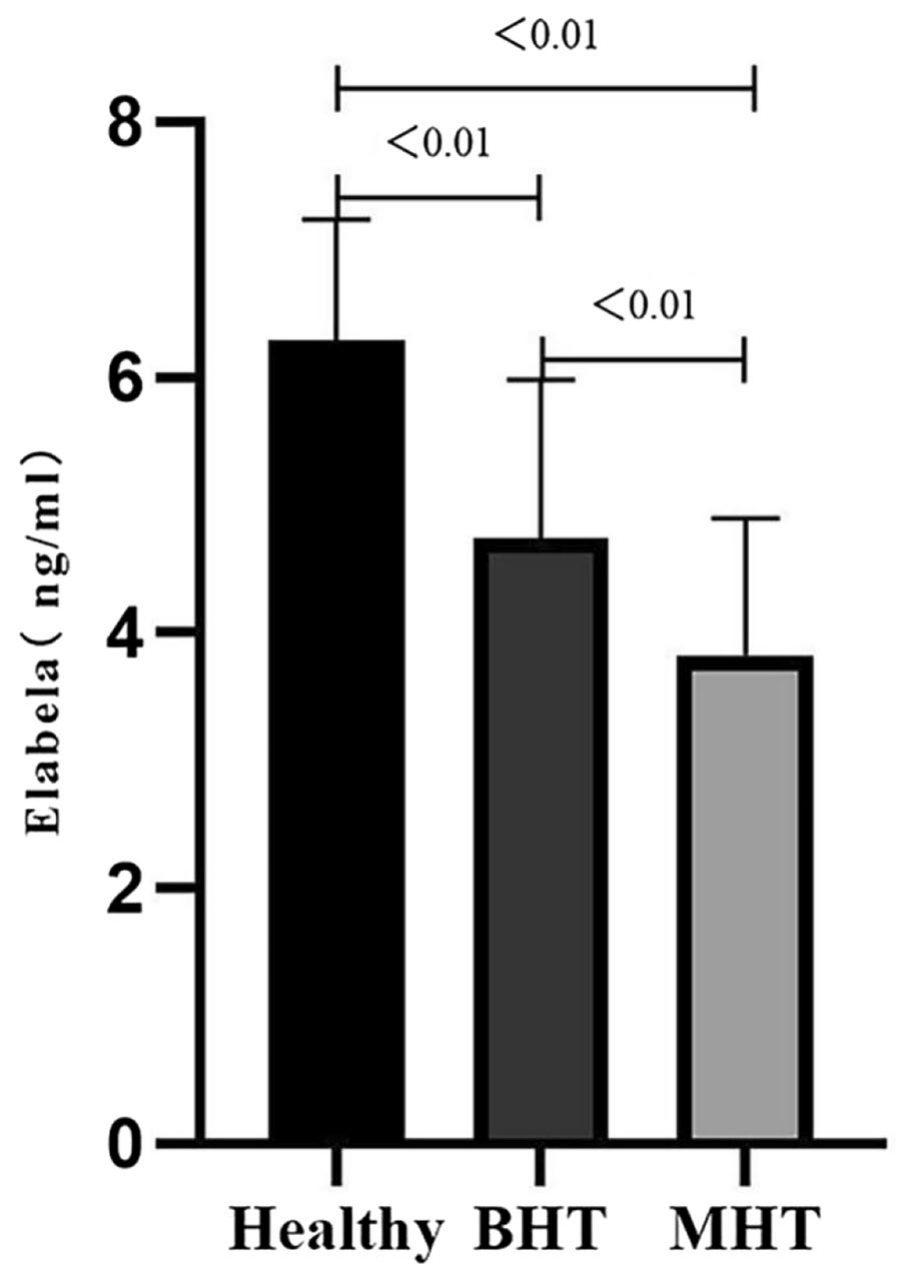
Serum Elabela levels in each group

Figure 1 shows that the serum Elabela levels gradually declined in MHT and BHT compared with the healthy control group (3.82 ± 1.08 vs. 4.73 ± 1.25 vs. 6.29 ± 0.94, respectively, *P* < 0.01). Pairwise comparisons were statistically significant (*P* < 0.05) (Fig. 1).

### Correlation of Elabela with variables

The Elabela levels were negatively correlated with BMI (*R* =  − 0.27, *P* = 0.02), SBP (R =  − 0.64, *P* < 0.01), DBP (*R* =  − 0.58, *P* < 0.01), uric acid (*R* =  − 0.39, *P* < 0.01), BUN (*R* =  − 0.53, *P* < 0.01), and Scr (*R* =  − 0.53 *P* < 0.01) but positively correlated with eGFR (*R* = 0.54, *P* < 0.01) (Table 2). However, no statistically significant correlation was observed between the Elabela and renal histopathological characteristics (Table 2, *P* > 0.05). A stepwise multiple linear regression analysis was performed with Elabela as the dependent variable by including the parameters that were significant in the correlation analysis. Additionally, SBP was closely and negatively correlated with the serum Elabela levels, and the linearity was up to 0.635 (Table 3).

**Table 2:** Correlation between Elabela and study variables

	R/Rs	*P*
Age	− 0.10	0.38
BMI (kg/m^2^)	− 0.28	0.02
SBP	− 0.64	< 0.01
DBP	− 0.58	< 0.01
Uric acid	− 0.39	< 0.01
BUN	− 0.53	< 0.01
Scr	− 0.53	< 0.01
eGFR	0.54	< 0.01
Histopathological		
TAIF	− 0.15	0.30
Intimal fibrosis	− 0.24	0.10
Myointimal cell proliferation	− 0.26	0.07

*SBP* systolic blood pressure, *DBP* diastolic blood pressure, *BUN* blood urine nitrogen, *Scr* serum creatinine, *eGFR* estimated glomerular filtration rate, *TAIF* tubular atrophy/interstitial fibrosis

**Table 3:** Stepwise multiple linear regression analysis of Elabela

	Unstandardized regression coefficients	SE	Standardized regression coefficients	*t*	*P*	95% CI of regression coefficients
Constant	9.287	0.631		14.708	$<$ 0.01	(8.028, 10.55)
SBP	− 0.028	0.004	− 0.635	− 7.029	$<$ 0.01	(− 0.036, − 0.020)

*SBP* systolic blood pressure, *SE* standard error, *CI* confidence interval, multiple R-squared 0.315, adjusted R-squared,0.305

SBP was negatively correlated with eGFR (*R* =  − 0.78, P < 0.01) and Elabela (*R* =  − 0.64, P < 0.01) but was positively correlated with uric acid (*R* =  − 0.53, *P* < 0.01), BUN (*R* = 0.68, *P* < 0.01), Scr (*R* = 0.78, *P* < 0.01), TAIF (*R* = 0.46, *P* < 0.01), intimal fibrosis (*R* = 0.49, *P* < 0.01), and myointimal cell proliferation (*R* = 0.79, *P* < 0.01).

## Discussion

This study revealed that serum Elabela levels were decreased in patients in the MHT and BHT groups compared with those in the healthy control, and lower Elabela levels were significantly negatively associated with SBP.

Apelin and APJ exist widely in kidneys and cardiovascular organs, and participate in physiological and pathological processes, such as vasodilation, reduction of vascular resistance, regulation of energy metabolism, and body fluid balance [23–25]. Previous studies have reported the effect of apelin in lowering blood pressure by relaxing arteries in animal models and humans [26, 27]. Other clinical studies have also confirmed that a decrease in the circulating apelin levels in the body is associated with an increased risk of hypertension [28, 29], whereas there is no significant correlation between receptor APJ and the risk of hypertension [30]. Elabela and apelin share APJ receptors, and perhaps they are similar in lowering blood pressure and protecting target organs. Recently, some studies have shown that Elabela relaxes contracted aortic vessels in a dose-dependent manner when combined with APJ in mice [15]. Moreover, it has been experimentally demonstrated that injecting Elabela into hypertensive rat and mouse models dilates their blood vessels, thus confirming the potential of Elabela to reduce blood pressure [31, 32]. On the one hand, injecting Elabela into hypertensive rats induced by a high-salt diet for 3 months relaxes vascular endothelium and reduces blood pressure. On the other hand, it downregulates renal fibrosis-related genes, improves renal interstitial fibrosis, and reduces glomerular and tubulointerstitial injuries [16].

The serum Elabela levels were measured in the 50 patients with MHT (*n* = 25) and BHT (*n* = 25) and 25 healthy controls. Notably, the levels of Elabela in patients with MHT and BHT were lower than those in healthy participants, especially in patients with MHT. Therefore, with the continuous increase in blood pressure, the level of Elabela decreased gradually, suggesting that the level of Elabela may be related to the progression of hypertension. In addition, Li et al. confirmed that the level of plasma Elabela decreased in patients with essential hypertension in a study of hypertension and Elabela [33], This result was consistent with those of our study.

Hypertension is an independent risk factor for the progression of chronic kidney diseases and also an important cause of end-stage renal diseases [34, 35]. The most common organ involved in MHT is the kidney [36], and the pathological feature of MHT is the injury of small blood vessels [37]. MHT has a variety of effects on the kidney, from apparently elevated serum creatinine to acute renal failure [38], whereas Elabela is primarily expressed in the kidney in adulthood [14]. We noticed, via pairwise comparisons, that serum Elabela levels exhibited a significant negative correlation with SBP, DBP, BUN, and Scr and a positive correlation with eGFR. Furthermore, the stepwise multiple linear regression analysis showed that SBP was the most relevant variable to lower the Elabela level. Elabela reduced blood pressure and protected the kidney by binding to the APJ, phosphorylating extracellular signal-regulated kinase (ERK), and inhibiting ERK activity on the NO/cGMP pathway, thereby reducing angiotensin II release and blocking the renin-angio-tensin system [39]. The results of this study also revealed that, as the systolic blood pressure increased, damage to the vascular endothelium (the more significant the myointimal cell proliferation) and kidney (the decrease in glomerular filtration rate) became more significant and serum Elabela level decreased. Perhaps, this may explain the decrease in Elabela in this study attributed to kidney and vascular injuries caused by a continuous increase in blood pressure. The major determining factor in the prognosis of MHT is the severity of renal impairment, and kidney failure is the leading cause of death in patients with MHT [7]. Previous studies have shown that the degree of renal damage due to persistently elevated blood pressure is proportional to the degree of exposure of renal microvessels to arterial pressure exposure. When afferent arterioles are undamaged, the transmission of intermittent or persistent arterial pressures to glomerular capillaries is significantly blocked. This is regulated by the self-protective effect of the kidney. When intermittent or persistent arterial pressures increase more significantly and exceed the range of self-regulatory protection, such as MHT, the glomerular capillary pressure increases significantly, decreasing the eGFR, which is characterized by myointimal cell proliferation and fibrinoid necrosis of human glomerular arterioles [40]. The mechanism of renal self-protection may explain the fact that the pathological damage of a hypertensive kidney has no significant correlation with the level of variable Elabela, unlike with SBP. Based on the aforementioned data, the level of Elabela may not be directly related to hypertensive renal damage. However, because SBP is an independent risk factor for Elabela level, the persistent increase in SBP aggravates renal damage and endothelial injury while simultaneously decreasing Elabela level.

This study has some limitations. First, it was not possible to explain whether the changes in the Elabela levels in each group were the cause or result of a sustained increase in blood pressure. Second, the number of patients in the experimental group was small. Therefore, the correlation between Elabela level and the pathological degree of renal damage in patients with hypertensive renal damage could not be accurately evaluated.

## Conclusions

Our study showed that the level of serum Elabela in patients with hypertension was decreased, and the level of Elabela in patients with MHT renal damage was lower than that in patients with BHT renal damage. Moreover, SBP was an independent risk factor for a decrease in Elabela level, which was significantly negatively correlated with SBP. Due to the small number of cases in this study and the single-center nature, there may be some bias in the results. The kidney is the most vulnerable organ, and by measuring the concentration of Elabela in hypertensive patients, it is possible to predict the extent of hypertension-associated kidney damage. At present, there are many studies on Elabela and hypertension-related kidney damage, but the specific mechanism is still not conclusive. In the future, we will continue to study the role of Elabela in hypertension-related kidney damage to clarify the possibility of using Elabela as a marker for predicting hypertensive kidney damage.

## Data Availability

The authors confirm that the datasets supporting the findings of this study are available from the corresponding author upon reasonable request.

## Abbreviations

APJ: Apelin receptor
BHT: Benign hypertension
BUN: Blood urea nitrogen
BMI: Body mass index
DBP: Diastolic blood pressure
eGFR: Estimated glomerular filtration rate
ESC: European Society of Cardiology
ESH: European Society of Hypertension
ERK: Extracellular signal-regulated kinase
MHT: Malignant hypertension
Scr: Serum creatinine
SBP: Systolic blood pressure
